# Snakebite Mortality in India: A Nationally Representative Mortality Survey

**DOI:** 10.1371/journal.pntd.0001018

**Published:** 2011-04-12

**Authors:** Bijayeeni Mohapatra, David A. Warrell, Wilson Suraweera, Prakash Bhatia, Neeraj Dhingra, Raju M. Jotkar, Peter S. Rodriguez, Kaushik Mishra, Romulus Whitaker, Prabhat Jha, for the Million Death Study Collaborators

**Affiliations:** 1 Shri Ramachandra Bhanj Medical College, Cuttack, Orissa, India; 2 Nuffield Department of Clinical Medicine, University of Oxford, Oxford, United Kingdom; 3 Australian Venom Research Unit, University of Melbourne, Melbourne, Australia; 4 Centre for Global Health Research (CGHR), Li Ka Shing Knowledge Institute, St. Michael's Hospital and Dalla Lana School of Public Health, University of Toronto, Toronto, Ontario, Canada; 5 Indian Institute of Health and Family Welfare, Hyderabad, India; 6 National AIDS Control Organization, New Delhi, India; 7 St. John's Research Institute, Bangalore, India; 8 Madras Crocodile Bank Trust and Centre for Herpetology, Chennai, India; Ghana Health Service, Ghana

## Abstract

**Background:**

India has long been thought to have more snakebites than any other country. However, inadequate hospital-based reporting has resulted in estimates of total annual snakebite mortality ranging widely from about 1,300 to 50,000. We calculated direct estimates of snakebite mortality from a national mortality survey.

**Methods and Findings:**

We conducted a nationally representative study of 123,000 deaths from 6,671 randomly selected areas in 2001–03. Full-time, non-medical field workers interviewed living respondents about all deaths. The underlying causes were independently coded by two of 130 trained physicians. Discrepancies were resolved by anonymous reconciliation or, failing that, by adjudication.

A total of 562 deaths (0.47% of total deaths) were assigned to snakebites. Snakebite deaths occurred mostly in rural areas (97%), were more common in males (59%) than females (41%), and peaked at ages 15–29 years (25%) and during the monsoon months of June to September. This proportion represents about 45,900 annual snakebite deaths nationally (99% CI 40,900 to 50,900) or an annual age-standardised rate of 4.1/100,000 (99% CI 3.6–4.5), with higher rates in rural areas (5.4/100,000; 99% CI 4.8–6.0), and with the highest state rate in Andhra Pradesh (6.2). Annual snakebite deaths were greatest in the states of Uttar Pradesh (8,700), Andhra Pradesh (5,200), and Bihar (4,500).

**Conclusions:**

Snakebite remains an underestimated cause of accidental death in modern India. Because a large proportion of global totals of snakebites arise from India, global snakebite totals might also be underestimated. Community education, appropriate training of medical staff and better distribution of antivenom, especially to the 13 states with the highest prevalence, could reduce snakebite deaths in India.

## Author Summary

Earlier hospital based reports estimate about 1,300 to 50,000 annual deaths from snakebites per year in India. Here, we present the first ever direct estimates from a national mortality survey of 1.1 million homes in 2001–03. Full-time, non-medical field workers interviewed living respondents about all deaths. The underlying causes were independently coded by two of 130 trained physicians. The study found 562 deaths (0.47% of total deaths) were assigned to snakebites, mostly in rural areas, and more commonly among males than females and peaking at ages 15–29. Snakebites also occurred more often during the rainy monsoon season. This proportion represents about 45,900 annual snakebite deaths nationally (99% CI 40,900 to 50,900) or an annual age-standardised rate of 4.1/100,000 (99% CI 3.6–4.5), with higher rates in rural areas (5.4) and with the highest rate in the state of Andhra Pradesh (6.2). Annual snakebite deaths were greatest in the states of Uttar Pradesh (8,700), Andhra Pradesh (5,200), and Bihar (4,500). Thus, snakebite remains an underestimated cause of accidental death in modern India, causing about one death for every two HIV-related deaths. Because a large proportion of global totals of snakebites arise from India, global snakebite totals might also be underestimated. Effective interventions involving education and antivenom provision would reduce snakebite deaths in India.

## Introduction

Alexander the Great invaded India in 326 BC, and was greatly impressed by the skill of Indian physicians; especially in the treatment of snakebites [1]. Since then, India has remained notorious for its venomous snakes and the effects of their bites. With its surrounding seas, India is inhabited by more than 60 species of venomous snakes – some of which are abundant and can cause severe envenoming [2]. Spectacled cobra (*Naja naja*), common krait *(Bungarus caeruleus)*, saw-scaled viper *(Echis carinatus)* and Russell's viper (*Daboia russelii*) have long been recognised as the most important, but other species may cause fatal snakebites in particular areas, such as the central Asian cobra (*Naja oxiana*) in the far north-west, monocellate cobra (*N. kaouthia*) in the north-east, greater black krait (*B. niger*) in the far north-east, Wall's and Sind kraits (*B. walli* and *B. sindanus*) in the east and west and hump-nosed pit-viper (*Hypnale hypnale*) in the south-west coast and Western Ghats [2].

Joseph Fayrer of the Indian Medical Service first quantified human snakebite deaths in 1869 for about half of “British India” (including modern Pakistan, Bangladesh and Burma), finding that 11,416 people had died of snakebites [3]. Subsequent estimates of human deaths from snakebite prior to Indian Independence ranged from 7,400 to 20,000 per year [4]–[6]. Government of India hospitals from all but six states reported only 1,364 snakebite deaths in 2008 [7] but this is widely believed to be an under-report as many victims of snakebite choose village-based traditional therapists and most die outside government hospitals. Community-based surveys in some localities have shown much higher annual mortality rates, ranging widely from 16.4 deaths/100,000 in West Bengal [8] to 161/100,000 in the neighbouring Nepal Terai [9]. However, such focal data cannot be extrapolated to provide national or even state totals because of the heterogeneity of snakebite incidence. These uncertainties have resulted in indirect estimates of annual snakebite mortality in India that varied from approximately 1,300 to 50,000 [6], [7], [10]–[13].

To fill this gap in knowledge, we estimated snakebite deaths directly from a large continuing study of mortality in India.

## Methods

### Ethics Statement

Ethics approval for the Million Deaths Study (MDS) was obtained from the Post Graduate Institute of Medical Research, St. John's Research Institute and St. Michael's Hospital, Toronto, Ontario, Canada [14]–[15].

Most deaths in rural India take place at home without prior attention by any qualified healthcare worker, so most causes are not medically certified [14]–[15]. Other approaches are therefore needed to help determine the probable causes of such deaths. The Registrar General of India (RGI) organises the Sample Registration System (SRS), which monitors all births and deaths in a nationally representative selection of 1.1 million homes throughout all 28 states and seven union territories of India. India was divided into approximately one million areas for the 1991 census, each with about 1,000 inhabitants. In 1993, the RGI randomly selected 6,671 of these areas to be represented in the SRS. Household characteristics were recorded and then enumerated twice yearly thereafter, documenting new births and deaths, but not the causes of death [16].

Since 2002, one of 800 non-medical field staff (trained by the RGI in appropriate fieldwork methods) visited each SRS area every six months to record a written narrative (in the local language) for each death from families or other reliable informants. In addition to the narratives, answers to standard questions about the deaths were also recorded in the field report. Fieldwork quality control methods were used routinely, including random re-sampling by teams reporting directly to the study investigators [14], [15]. This survey is part of the MDS, which seeks to assign causes to all deaths in SRS areas for the period between 2001–14 [14]–[16], [17]–[19].

These field reports, or ‘verbal autopsies’, were emailed randomly (based on the language of the narrative) to at least two of 130 collaborating physicians trained in disease coding. Physicians worked independently to assess the probable underlying cause of death, assigning each case a three-character International Classification of Diseases (ICD; 10^th^ revision) code [20]. Any differences between the two coders were resolved by anonymous reconciliation between them (asking each to reconsider) or, for persisting differences, adjudication by a third physician (3% or 15/562 of snakebite deaths, and 18% or 22,845/122,848 of all deaths). The physician coders' training and their written guidelines (available online [21]) instructed them to use their best medical judgement to determine the most probable cause of death. Field reports could not be collected on 12% of the identified deaths due to migration or change of residence. As these missing deaths were mostly random, a systematic misclassification in cause of death was unlikely. We used logistic regression to quantify the odds of snakebite versus other deaths for gender, state, religion, education, occupation, place of death and season. Risk is measured compared to the reference group of lowest risk for each variable. Climate data on rainfall and temperature were obtained for each state from the India Meteorological Department [22]–[23].

The proportion of cause specific deaths in each age category was weighted by the inverse probability of household selection within rural and urban sub divisions of each state, to account for the sampling design [16]. Using methods described earlier [14]–[15], [17]–[19], the resulting proportion of deaths from each cause was applied to the United Nations (UN) population division estimates of deaths in India in 2005 [24] (9.8 million, upper and lower limits 9.4–10.3 million) to generate cause- specific death totals and rates. The UN totals (which undergo independent demographic review [24]) were used because the SRS underestimates adult mortality rates by about 10% [25]–[26]. The UN totals are not affected by the 12% of the SRS-enumerated deaths that were unavailable for interview in our survey. Totals for 2005 were used because they: (i) were most complete; (ii) could be compared to the available Indian Census projections for 2006; and (iii) captured information prior to the implementation of a new national health program in rural areas [27]. However, applying the 2001–03 proportions to the 2005 total deaths did not introduce major biases since there was little change in the yearly distribution in snakebite deaths in our survey, or in the annual number of deaths reported from snakebites in routine national hospital surveillance data between 2003 and 2008 [7].

## Results

### Snakebite deaths in study and nationally

Of the 643 deaths coded by physicians as ICD-10 codes X20–X29 (contact with venomous animals and plants), 523 (81%) were coded as X20 (venomous snakes) and review of these yielded no misclassified causes. Central re-examination of the symptoms and key words found 39 of 45 deaths coded as X27 (animals) and X29 (uncertain) to be snakebite deaths. We excluded 75 deaths coded as X21–X25 (various arthropods), X26 (marine organisms) and X28 (plants).

Among all 122,848 deaths, 2,179 of the deaths that were randomly chosen to be re-interviewed by independent teams were eventually matched to the identical houses and individuals of the MDS. Of the 2,179 re-sampled deaths, 9 were coded as snakebites, and 7 of these were found in the MDS. Thus, the sensitivity and specificity of the SRS field survey, assuming the re-sample deaths are the standard comparison, was 78% (7/9) and 100% (2,170/2,170), respectively.

A total of 562 of the 122,848 deaths (0.47% weighted by sampling probability or 0.46% unweighted) were from snakebites (Table 1). Almost all snakebite deaths (544 or 97%) were in rural areas. More men (330, 59%) than women (232, 41%) died from snakebites (overall ratio of 1.4 to 1). The proportion of all deaths from snakebites was highest at ages 5–14 years. Only 23% (127/562) of the deaths occurred in a hospital or other healthcare facility.

**Table 1:** Snakebite deaths in the present study, 2001–03 and estimated national totals, by age.

	Study deaths 2001–03				All India estimates 2005				
	Numbers attributed		Proportion snakebite deaths per 1,000*	Died in health facility	Rural area	All causes deaths/population (million): UN estimates †	Snakebite deaths in thousands	Death rate per 100,000	
Age in years	Male/Female	Snakebite/all causes						National	Rural
0–4	29/23	52/23,630	2.1	8	52	2.3/128	5.0	3.9	4.9
5–14	73/41	114/3,881	28.5	24	111	0.3/246	9.7	4.0	5.1
15–29	80/62	142/9,121	15.9	31	134	0.7/313	11.0	3.5	4.7
30–44	60/44	104/10,872	9.4	30	102	0.9/222	8.3	3.8	5.3
45–59	52/27	79/18,133	4.6	22	73	1.5/142	6.8	4.8	6.2
60–69	21/24	45/21,136	2.2	6	44	1.5/49	3.3	6.6	8.7
70+	15/11	26/36,075	0.7	6	28	2.6/30	1.8	6.2	8.0
**All ages**	**330/232**	**562/122,848**	**4.7**	**127 (23%)**	**544 (97%)**	**9.8/1,130**	**45.9**	**4.1**	**5.4**
**(99% CI )**							**(40.9, 50.9)**	**(3.6, 4.5)**	**(4.8,6.0)**

The overall study death total of 122,848 includes 8.7% senility, unspecified or ill defined deaths, which were not assigned to any specific disease categories.

*Proportional snakebite mortality per 1,000 after applying sample weights to adjust urban-rural probability of selection.

**†:** United Nations 2005 estimates for India.

Expressed as national totals, snakebites caused 45,900 deaths in India in 2005 (99% CI 40,900 to 50,900). The age-standardised death rate per 100,000 population per year was 4.1 (99% CI 3.6–4.5) nationally and was 5.4 (99% CI 4.8–6.0) in rural areas.

### Risk factors and seasonality

**Figure 1:**
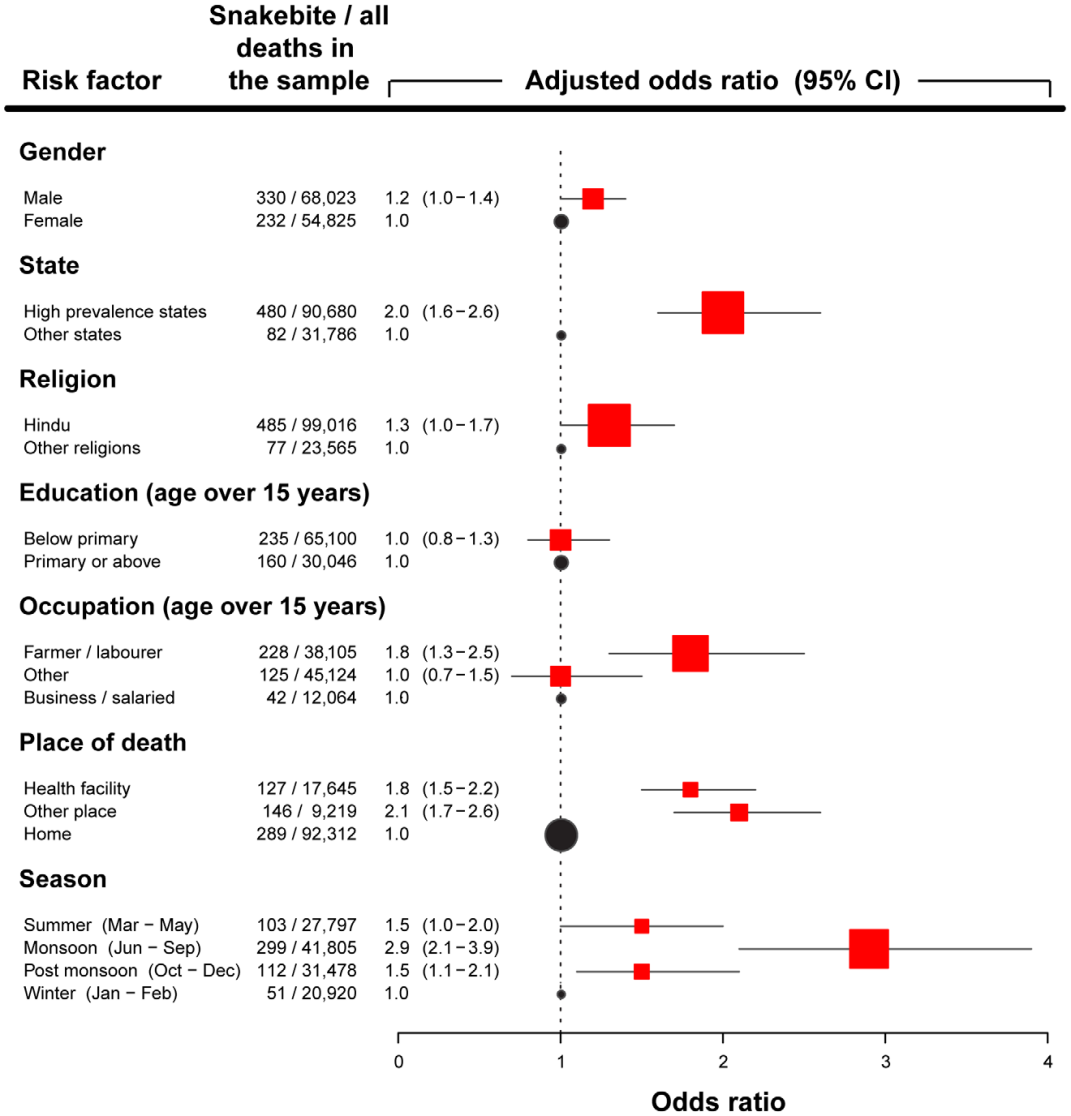
Selected risk factors for snakebite mortality in India (study deaths 2001–03). Odds ratio after adjusting for age, gender and states with a high prevalence of snakebite deaths (see definition in Table 2). Occupation ‘Other’ includes students and house wives.

**Figure 2:**
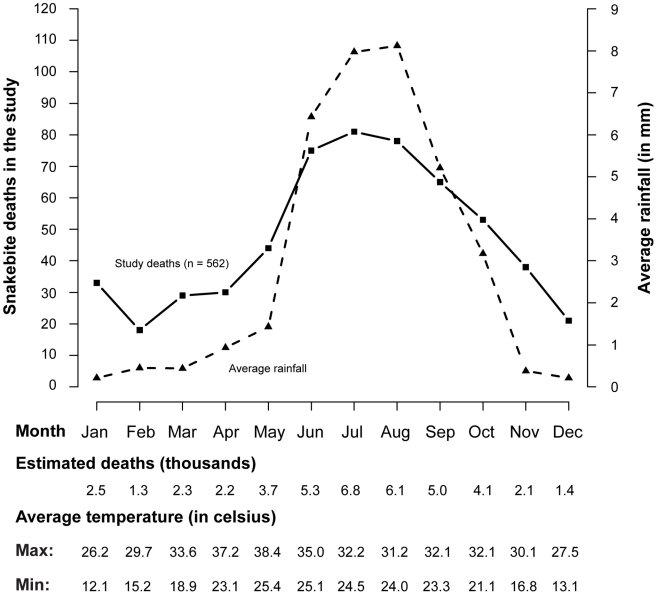
Seasonality pattern of snakebite mortality and rainfall in states with high prevalence of snakebite deaths (2001–03). Rainfall amount (mm) is cumulative daily rainfall for the past 24 hours measured by the India Meteorological Department [22], [23]. Maximum and minimum temperatures are also measured daily and presented as monthly averages across the 13 snakebite high prevalence states. Pearson correlation coefficients between snakebite mortality and weather were: (i) rainfall; 0.93 (p<0.0001); (ii) minimum temperature: 0.80 (p = 0.0017); (iii) maximum temperature: 0.35 (p = 0.2585).

Figure 1 shows the odds ratios for snakebite deaths versus other deaths, adjusted for age, gender, and for high prevalence states (13 states with age-standardised snakebite death rates greater than 3 per 100,000) versus other states. The risks of snakebite deaths were significantly increased among Hindus and farmers/labourers, deaths occurring outside home, and during the monsoon months of June to September (Figures 1 and 2). In contrast, gender and education were not significantly associated with risk of snakebite death. About 5,000–7,000 snakebite deaths per month occurred during the monsoon period, compared to less than 2,000 deaths in the winter months. Monthly numbers of snakebite deaths correlated with rainfall (R = 0.93, p<.0001) and mean minimum temperature (R = 0.80, p = 0.0017), but not with mean maximum temperature (R = 0.35, p = 0.2585; Figure 2).

### State mortality patterns

**Figure 3:**
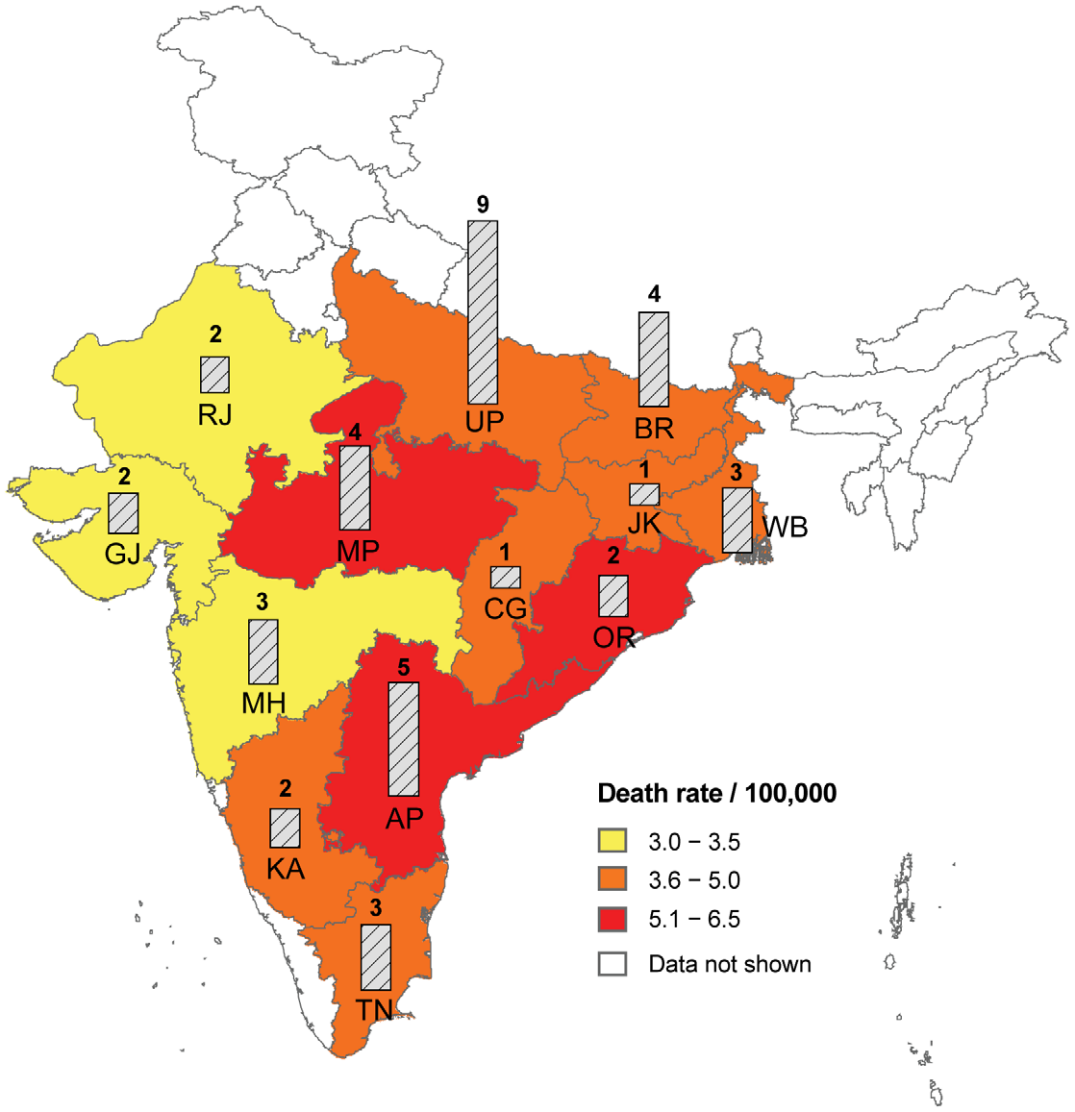
Estimated deaths and standardized death rates in states with high prevalence of snakebite deaths, 2005. Death rates are standardised to 2005 UN population estimates for India [24]. The vertical bars represent the state wise estimated deaths (in thousands). Total snakebite deaths for the 13 states with high-prevalence of snakebite death are 42,800 or 93% of the national total (these states have about 85% of the total estimated population of India). States where the snakebite death rate was below 3/100,000 or where populations are less than 10 million are not shown. The states with high-prevalence of snakebite deaths are: AP-Andhra Pradesh, BR-Bihar, CG-Chhattisgarh, GJ-Gujarat, JH-Jharkhand, KA-Karnataka, MP Madhya Pradesh, MH-Maharashtra, OR-Orissa, RJ- Rajasthan, TN-Tamil Nadu, UP-Uttar Pradesh, WB-West Bengal.

Annual age-standardised mortality rates per 100,000 from snakebite varied between states, from 3.0 (Maharashtra) to 6.2 (Andhra Pradesh) in the 13 states with highest prevalence (average 4.5) compared to 1.8 in the rest of the country (Table 2; Figure 3). Total deaths were highest in Uttar Pradesh (8,700), Andhra Pradesh (5,200), and Bihar (4,500). The age and gender of snakebite deaths also varied by region, although these differences were not significant due to the small numbers of snakebite deaths in each state. Deaths at ages 5–14 years were prominent in the states of Jharkhand and Orissa, whereas deaths at older ages were prominent in Andhra Pradesh, Bihar, Madhya Pradesh, and Uttar Pradesh (data not shown). In Bihar, Madhya Pradesh, Maharashtra and Uttar Pradesh, female deaths exceeded male deaths (Table 2).

**Table 2:** Estimated snakebite deaths in the Indian states with a high prevalence of snakebite deaths, 2005.

	Study deaths 2001–03				Estimated state and national deaths 2005	
State	Snakebite/all causes	Male/female	Died outside health facility	Proportional mortality/1,000	Snakebites deaths in thousands	Death rate per 100,000
**States with high-prevalence of snakebite deaths** *						
Andhra Pradesh	45/5,831	31/14	42	7.4	5.2	6.2
Madhya Pradesh	41/7,257	20/21	31	5.7	4.0	5.9
Orissa	37/7,364	22/15	26	5.2	2.2	5.6
Jharkhand	12/2,179	8/4	12	5.8	1.5	4.9
Bihar	50/9,824	21/29	45	5.8	4.5	4.9
Tamil Nadu	38/6,316	26/12	28	5.1	3.1	4.7
Uttar Pradesh	78/15,403	36/42	72	4.8	8.7	4.6
Chhattisgarh	13/2,328	6/7	11	4.6	1.0	4.4
Karnataka	41/6,961	32/9	32	5.0	2.4	4.2
West Bengal	40/8,330	24/16	20	4.7	3.0	3.5
Gujarat	28/6,151	20/8	20	4.1	1.9	3.5
Rajasthan	29/6,769	18/11	24	4.2	2.1	3.3
Maharashtra	28/6,274	9/19	18	3.9	3.2	3.0
**Sub total**	**480/90,987**	**273/207**	**381**	**5.1**	**42.8**	**4.5**
**Remaining states**	82/31,861	57/25	54	2.2	3.1	1.8
**All India**	**562/122,848**	**330/232**	**435**	**4.7**	**45.9**	**4.1**
**(99% CI)**					**(40.9, 50.9)**	**(3.6, 4.5)**

States are listed in descending order of death rates. Death rates are standardised to 2005 UN national estimates for India.

*States with a high-prevalence of snakebite deaths are defined as those with more than 10 million people where the annual snakebite death rate exceeds 3 per 100,000 population.

## Discussion

Snakebite remains an important cause of accidental death in modern India, and its public health importance has been systematically underestimated. The estimated total of 45,900 (95% CI 40,900–50,900) national snakebite deaths in 2005 constitutes about 5% of all injury deaths and nearly 0.5% of all deaths in India. It is more than 30-fold higher than the number declared from official hospital returns [7]. The underreporting of snake bite deaths has a number of possible causes. Most importantly, it is well known that many patients are treated and die outside health facilities – especially in rural areas. Thus rural diseases, be they acute fever deaths from malaria and other infections [19] or bites from snakes or mammals (rabies; [28]), are underestimated by routine hospital data. Moreover, even hospital deaths may be missed or not reported as official government returns vary in their reliability, as shown from a study of snakebites in Sri Lanka [29]. The true burden of mortality from snakebite revealed by our study is similar in magnitude to that of some higher profile infectious diseases; for example, there is one snakebite death for every two AIDS deaths in India [18]. Consequently, snakebite control programmes should be prioritised to a level commensurate with this burden.

Our data suggest underestimation in recent global estimates of mortality from snakebite deaths [10]: the upper bounds of recent annual estimates were 94,000 deaths globally and 15,000 deaths in India. This total for India is only about one-third of the snake bite deaths detected in our study. The incidence of snakebite deaths per 100,000 population per year in a recent community-based study in Bangladesh was similar to ours [30], suggesting that much of South Asia might have thousands more snakebite deaths than is currently assumed. Considering the widely accepted gross underestimation of snakebite deaths in Africa [11], it seems highly probable that well over 100,000 people die of snakebite in the world each year.

A minimal number of non-fatal snakebites in India may be estimated with far less certainty. Indian data from routine public sector hospitals [7] are clearly under-reports of deaths (recording only 1 in 5 of the deaths we estimated to have occurred in hospital). Nonetheless, the ratio of non-fatal bites (about 140,000) to fatal bites (about 2,200) in these hospital data from 2003–08 (about 64∶1) is informative of the relative burden of bites to deaths. Very crudely, even if we halve the fatal/nonfatal bite ratio to 32, this would suggest at least 1.4 million non-fatal bites corresponding to the 45,000 fatal bites. The actual number of non-fatal bites in India may well be far higher, as the community-based study in Bangladesh found about 100 non-fatal bites for each death [30].

Our study has limitations; notably the misclassification of snakebite deaths. However, snakebites are dramatic, distinctive and memorable events for the victim's family and neighbours, making them more easily recognizable by verbal autopsy. We observed a reasonably high sensitivity and specificity when compared to re-sampled deaths. Confusion with arthropod bites and stings is unlikely because of the different circumstances, size and behaviour of the causative animal and the course of envenoming. For example, most deaths from hymenoptera stings result from rapidly evolving anaphylaxis. Kraits (important agents of snakebite death in South Asia) may unobtrusively envenom sleeping victims, who may die after developing severe abdominal pain, descending paralysis, respiratory failure and convulsions [31]. Such deaths might not be associated with snakebite at all. These examples suggest possible underestimation of deaths in our data.

Since the numbers of deaths observed in each state were small, the estimated totals for each state are uncertain. However, the state distribution is broadly consistent with that reported by the RGI survey of deaths in selected rural areas in the 1990s [32]. The marked geographic variation across states in our study is similar to that in a country-wide survey conducted during the period 1941–45, which identified Bengal, Bihar, Tamil Nadu, Uttar Pradesh, Madhya Pradesh, Maharashtra and Orissa as having the highest death rates from snakebite [6]. Moreover, despite the obvious underestimates in hospitalised data [7], their geographical distribution of bites and deaths were similar to what we observed from household reports of deaths. The marked differences in snakebite mortality between states of India may be attributable to variations in human, snake and prey populations, and in local attitudes [8] and health services. The 13 states with the highest snakebite mortality are inhabited by the four most common deadly venomous snakes: *Naja naja*, *Bungarus caeruleus*, *Echis carinatus* and *Daboia russelii*. With the exception of *E. carinatus*, which favours open wasteland, these are widely distributed species of the plains and low hills where most Indians live. While some species can inhabit altitudes of up to 2,700 metres [2], this is exceptional and higher mountainous regions have considerably lower death rates.

As found in an earlier study [33], the peak age group of snakebite deaths is 15–29 years (25% or 142/562). However, the relative risk of dying from snakebite versus another cause was greater at ages 5–14 years. The peak age range and gender associated with snakebite mortality varied between states, perhaps reflecting differences in the relative numbers of children and women involved in agricultural work [34]–[35]. The slight excess among Hindus may reflect more tolerance of snakes and greater use of traditional treatments [2]. Snakebites and snakebite fatalities peak during the monsoon season in India [33], [36] and worldwide [10], probably reflecting agricultural activity, flooding, increased snake activity, and abundance of their natural prey.

Only 23% of the snakebite deaths identified in our survey occurred in hospital, consistent with an earlier study from five states [33]. This emphasises three points: (i) hospital-based data reflect poorly the national burden of fatal snakebites; (ii) inadequacy of current treatment of snakebite in India; and (iii) vulnerability of snakebite victims outside hospital. Practicable solutions include strengthening surveillance to allow a more accurate perception of the magnitude of the problem, improving community education to reduce the incidence of snakebites and speed up the transfer of bitten patients to medical care, improving the training of medical staff at all levels of the health service (including implementation of the new WHO guidelines [12]), and deployment of appropriate antivenoms and other interventional tools where they are needed in rural health facilities to decrease case fatality [36]–[38]. In addition, phylogenetic and venom studies are needed to ensure appropriate design of antivenoms to cover the species responsible for serious envenoming.
